# Filter quality of electret masks in filtering 14.6–594 nm aerosol particles: Effects of five decontamination methods

**DOI:** 10.1371/journal.pone.0186217

**Published:** 2017-10-12

**Authors:** Tzu-Hsien Lin, Chih-Chieh Chen, Sheng-Hsiu Huang, Chung-Wen Kuo, Chane-Yu Lai, Wen-Yinn Lin

**Affiliations:** 1 Department of Dental Hygiene, China Medical University, Taichung, Taiwan; 2 Institute of Occupational Medicine and Industrial Hygiene, National Taiwan University, Taipei, Taiwan; 3 Institute of Environmental Engineering and Management, National Taipei University of Technology, Taipei, Taiwan; 4 Department of Occupational Safety and Health, Chung Shan Medical University, Taichung, Taiwan; 5 Department of Occupational Medicine, Chung Shan Medical University Hospital, Taichung, Taiwan; VIT University, INDIA

## Abstract

This study investigates the effects of five decontamination methods on the filter quality (*q*_*f*_) of three commercially available electret masks—N95, Gauze and Spunlace nonwoven masks. Newly developed evaluation methods, the overall filter quality (*q*_*f*,*o*_) and the *q*_*f*_ ratio were applied to evaluate the effectiveness of decontamination methods for respirators. A scanning mobility particle sizer is utilized to measure the concentration of polydispersed particles with diameter 14.6–594 nm. The penetration of particles and pressure drop (Δ*p*) through the mask are used to determine *q*_*f*_ and *q*_*f*,*o*_. Experimental results reveal that the most penetrating particle size (MPS) for the pre-decontaminated N95, Gauze and Spunlace masks were 118 nm, 461 nm and 279 nm, respectively, and the respective penetration rates were 2.6%, 23.2% and 70.0%. The Δ*p* through the pretreated N95 masks was 9.2 mm H_2_O at the breathing flow rate of heavy-duty workers, exceeding the Δ*p* values obtained through Gauze and Spunlace masks. Decontamination increased the sizes of the most penetrating particles, changing the *q*_*f*_ values of all of the masks: *q*_*f*_ fell as particle size increased because the penetration increased. Bleach increased the Δ*p* of N95, but destroyed the Gauze mask. However, the use of an autoclave reduces the Δ*p* values of both the N95 and the Gauze mask. Neither the rice cooker nor ethanol altered the Δ*p* of the Gauze mask. Chemical decontamination methods reduced the *q*_*f*,*o*_ values for the three electret masks. The value of *q*_*f*,*o*_ for PM_0.1_ exceeded that for PM_0.1–0.6_, because particles smaller than 100 nm had lower penetration, resulting in a better *q*_*f*_ for a given pressure drop. The values of *q*_*f*,*o*_, particularly for PM_0.1_, reveal that for the tested treatments and masks, physical decontamination methods are less destructive to the filter than chemical methods. Nevertheless, when purchasing new or reusing FFRs, penetration should be regarded as the priority.

## Introduction

Standard patient care requires sterilizing or disinfecting surgical equipment and reclaimed medical devices that are contaminated by infectious agents [1]. Those items do not include masks, which should not be reused under normal circumstances. Healthcare workers (HCWs) often use disposable masks following outbreaks of SARS [2–4], H1N1 [5,6] MERS and influenza. In hospitals, HCWs use these masks as the front-line defense against airborne droplets and bioaerosols; however, in public, most people use the masks as a precaution or from a sense of panic.

The supply of masks may not meet demand during outbreaks or pandemics of diseases that are spread by human-to-human transmission through infectious bioaerosols. Therefore, some health authorities have considered reusing masks after decontamination to remove any infectious material. Most health authorities do not recommend the reuse of masks, but several users have attempted to use various simple methods to reclaim them to protect against noninfectious ambient particulate matter (PM)—particularly PM_2.5_, PM_1_ or PM_0.1_. NIOSH published a series of research articles on mask decontamination [7–9]. The filtering effectiveness of reclaimed filtering facepiece respirators (FFRs) and masks varies among brands and decontamination methods, so relevant results are applicable only to FFRs treated under the same conditions. Therefore, further tests must be performed to evaluate the filtration performance of filters before and after reclamation. This investigation applied three types of disposable mask, N95 FFRs (Dust Respirator 8210; 3M, St. Paul, MN, N95), gauze double-layer electret masks (Gauze, commercialized type, China) and spunlace nonwoven masks (Spunlace, Oimo, Taiwan), all of which are frequently used, and which can be reused when hospitals in Taiwan have insufficient masks for frontline healthcare workers [4].

Effective reclamation of FFRs requires low penetration of sodium chloride (NaCl) particles, and low resistance to the flow of air through the filter [7,9]. The most frequently performed tests on filtration performance involve a polydispersed aerosol and evaluation of the most penetrating particle size (MPS) [10]. NaCl and dioctyl phthalate (DOP) are adopted as challenge aerosols with count median diameters (CMD) of 75 nm±20 nm and 185 nm±20 nm, and geometric standard deviations (GSD) of 1.86 and 1.6, respectively. ISO published test conditions include a numerical median particle size distribution (PSD) of between 60 nm and 100 nm, and electromobility diameters of 160nm and 210 nm for NaCl and liquid paraffin oil, respectively [11]. Both ISO conditions involve a polydispersed aerosol with a GSD of between 1.4 and 1.8. Calculations of penetration based on the published NIOSH and ISO criteria involve the penetration of particles of all sizes, instead of the penetration of particles of any specific size. The MPSs of various FFRs are not always within the standard range of CMD. Huang *et al*. observed that the MPS in electret filter media varies with face velocity, fiber diameter, packing density, filter thickness and charge density [12]. Current measurement methods can provide an accurate range of penetrating PSDs [13], and elucidate the penetration and filter quality for particles covering a wide range of particle sizes.

The filter quality *q*_*f*_ [12,14–16] is a possible criterion for comparing performance of filters before and after decontamination, and is the ratio of penetration indicator to the pressure drop. The *q*_*f*_ is also called the figure of merit (FOM) [17–20]. The best filter should give the highest collection efficiency with the lowest pressure drop, where a higher *q*_*f*_ indicates a better filter [15].

This work compares the filter qualities of three electret masks before and after the implementation of five decontamination methods that are frequently employed in Taiwan. The comparison is based on particle penetration measurements and the pressure drop through the mask. The filter quality and overall filter quality (integrated for PM_0.1_, PM_0.1–0.6_) were then calculated to measure the filtration performance.

## Materials and method

### Experimental procedure

**Fig 1:**
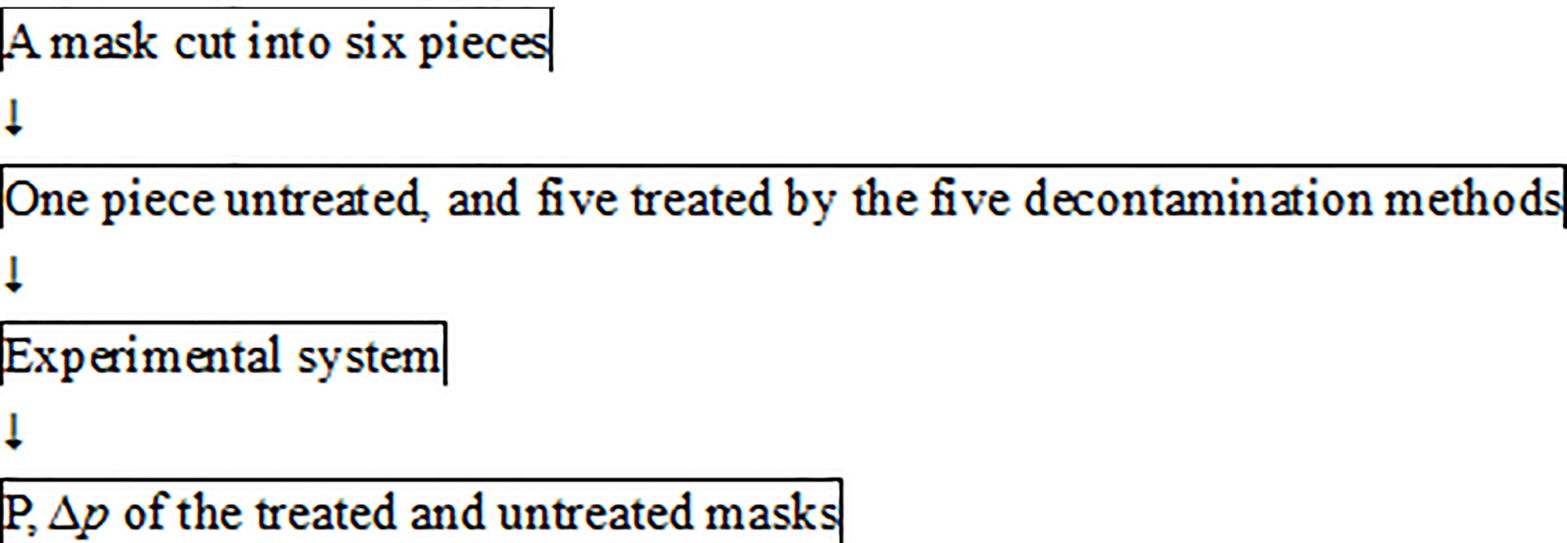
Experimental procedure.

Fig 1 illustrates the experimental procedure by which three types of electret mask (N95, Gauze, and Spunlace) were tested. Table 1 lists the five selected decontamination methods: (1) physical decontamination using a traditional electric rice cooker that was made in Taiwan to provide dry heat; (2) physical decontamination using an autoclave to provide moist heat, and low-temperature chemical decontamination using (3) ethanol, (4) isopropanol and (5) bleach. All of these methods are frequently adopted in hospitals in Taiwan.

**Table 1:** Decontamination methods.

Method	Experimental Conditions and Parameters
Rice cooker	Place the test masks in a traditional electric rice cooker using dry heat for 3 minutes (149~164°C, without adding water).
Autoclave	Set the temperature at 121°C with 1.06 kg cm^−2^ for 15 minutes.
Ethanol *	10 min submersion in 70% ethanol solution.
IPA *	10 min submersion in 100% isopropanol solution.
Bleach *	10 min submersion in 0.5% sodium hypochlorite solution (original concentration = 0.5% available as Cl_2_). Manufacturing specification: 0.5% (w/w) available chlorine.

*Liquid submersion methods. Following each exposure, masks were placed in a laboratory chemical hood and allowed to air-dry overnight before performing the laboratory aerosol filtration test.

### Experimental system

**Fig 2:**
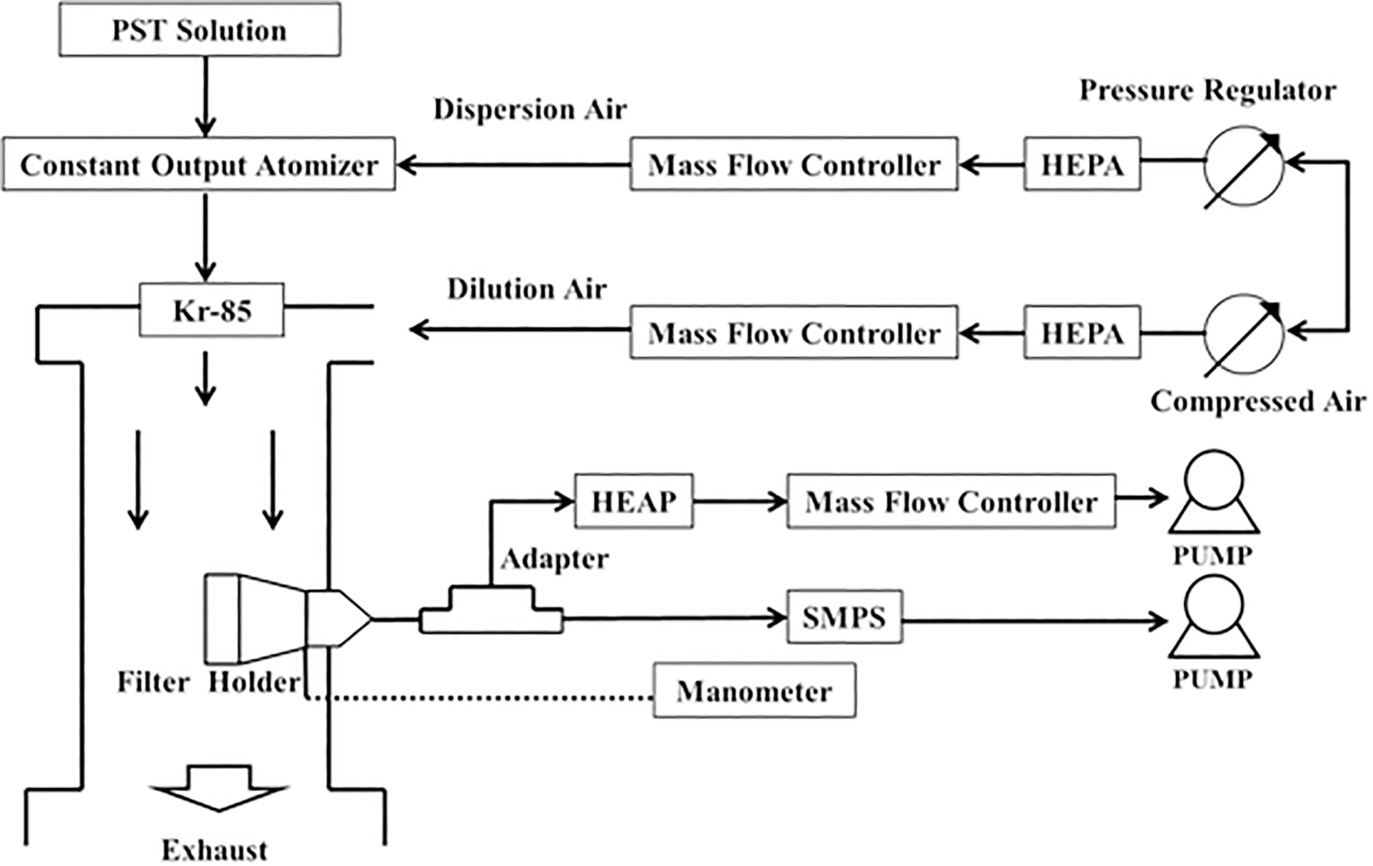
Experimental setup.

Fig 2 shows the measured penetration of particles and the pressure drop through the test mask. In the particulate penetration test, particles were formed using a constant-output atomizer (Model 3075, TSI Inc., St. Paul, MN) to atomize potassium sodium tartrate tetrahydrate (PST) solutions into polydispersed droplets [16]. The droplets were passed through a neutralizer that contained the radiation source Kr-85 (Model 3077, TSI Inc., St. Paul, MN), which neutralized them to the Boltzmann equilibrium state. Finally, the neutralized aerosols were passed into a test chamber, and diluted in filtered air. The overall quality factor was calculated over a wide range of sizes from 14.6nm to 594 nm which include viral particle size range. To obtain this size range, a challenge aerosol size distribution was utilized with a CMD of 101 nm±10 nm, and a GSD of 2.01±0.08.

The breathing flow rate of heavy-duty workers (85 L min^−1^) [21] was simulated by a calculated surface velocity of 8.3 cm s^−1^ over the whole mask, which value is close to that used elsewhere [16]. Six pieces with a pieces with diameter 41 mm were cut from a full mask in each batch test. Each piece included the original layers to fit the filter holder, providing an effective diameter of 39 mm and a calculated effective filtration area of 11.94 cm^2^. To yield the specified surface velocity, the flow rate of the vacuum pump connected to each filter holder was set to 5.95 L min^−1^, which was maintained using a mass flow controller. A scanning mobility particle sizer (SMPS, Model 3934, TSI Inc., St. Paul, MN) was used to measure the PSDs (14.6–594 nm) upstream and downstream of the test mask. The SMPS sampling period in each test was set to 300s upscan and 30s downscan. Each decontamination type test was performed six times.

To ensure the stability of the experimental system, all flow rates in the system were controlled and monitored with mass flow controllers (Hastings Instrument, Hampton, VA), and the flow rates were adjusted using an infrared bubble meter (Gillian Instrument Co. West Caldwell, NJ).

A manometer was used to measure the pressure drop (Δ*p*) through the test mask, and flow rates were set from 1.5 to 10 L min^−1^ using a mass flow controller.

### Data processing

The penetration (*P*, %) was derived using the equation,

$$P(\%)=\frac{C_{d}}{C_{u}}\times 100\%$$ (1)

where *C*_*d*_ and *C*_*u*_ denote the concentrations of particles of the same size downstream and upstream, respectively. The filter quality factor (*q*_*f*_, mm H_2_O^-1^) combines *P* and Δ*p* as follows [12,16].

qf(mmH2O−1)=ln⁡(1P)∆p (2)

The newly developed evaluation method—overall filter quality (*q*_*f*,*o*_, mm H_2_O^−1^) is obtained using the equation,

$$q_{f,o}(mm\;H_{2}O^{-1})=\sum_{i=14.6}^{594}q_{f}\times ∆log{dp}_{i}$$ (3)

### Statistical analysis

Experimental data were analyzed using SPSS software, version 17.0 (SPSS Inc., Chicago, IL). After linear regression between the Δ*p* and flow rate was performed, the assumption of homogeneity of the regression slopes was carried out to confirm the effects of decontamination using analysis of covariance (ANACOVA) [16]. The statistics were considered significant at *p*<0.05.

## Results and discussion

### Penetration of particles through mask before and after decontamination

**Fig 3:**
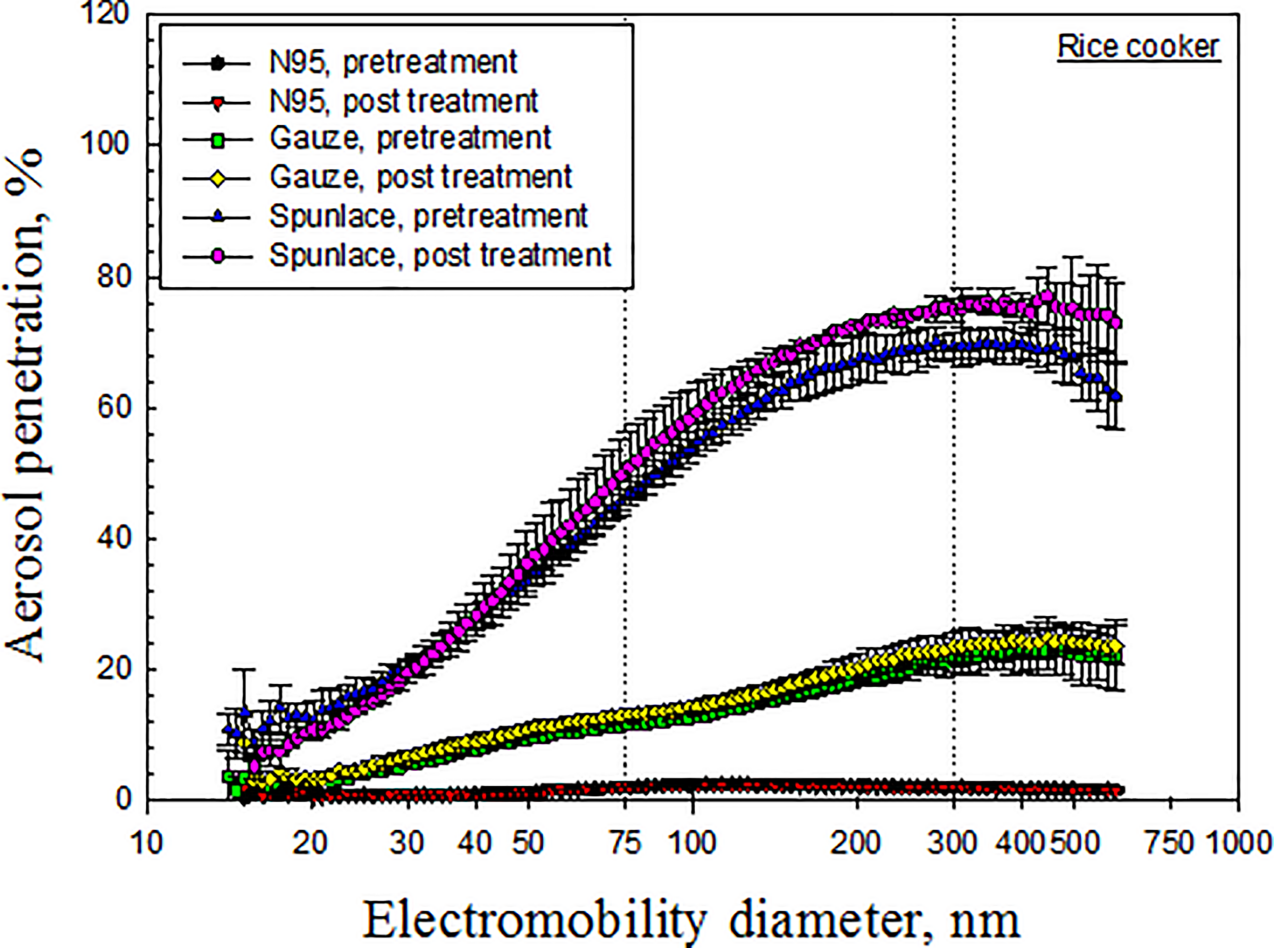
Penetration of particles through mask after decontamination using rice cooker.

**Fig 4:**
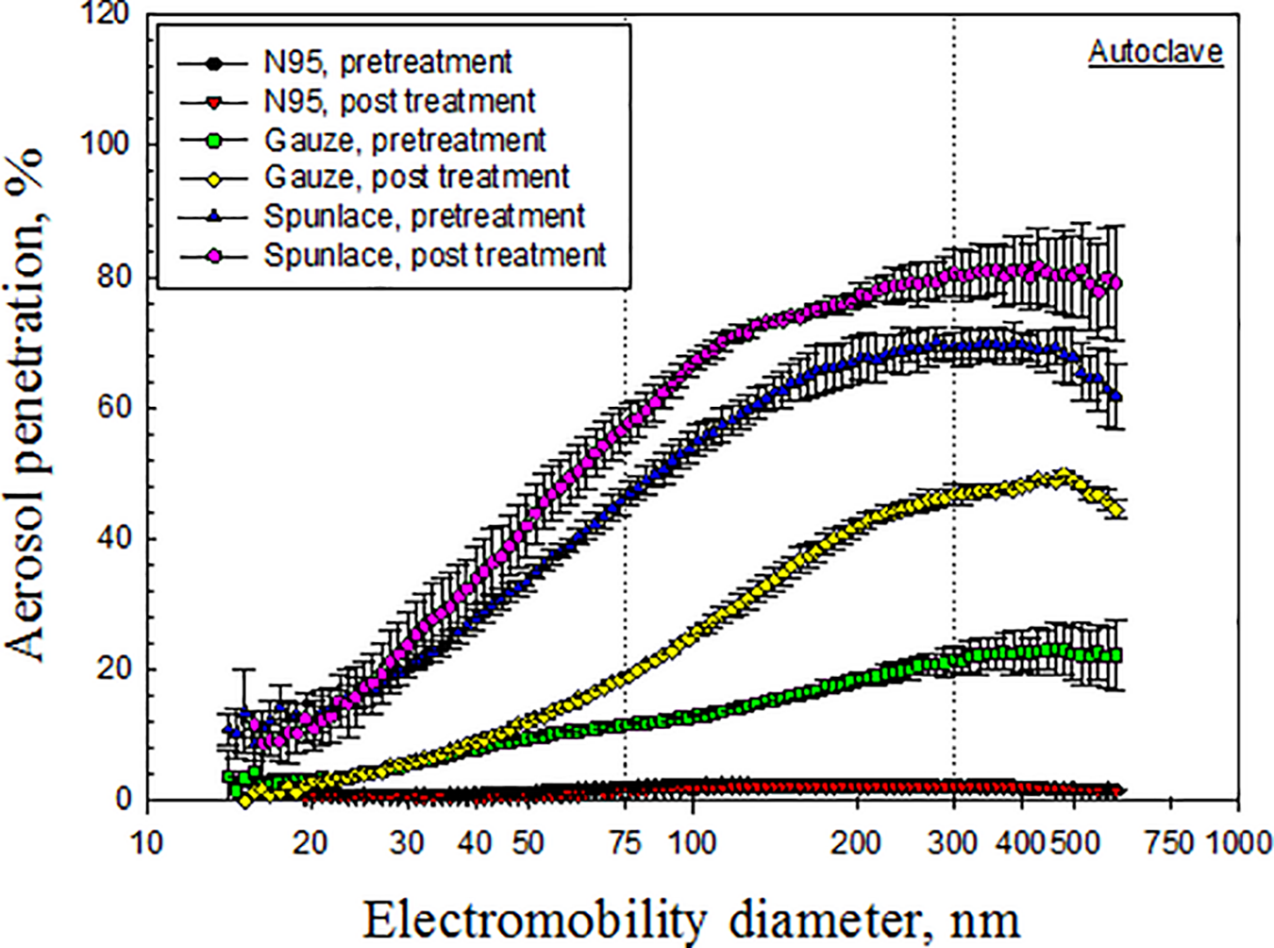
Penetration of particles through mask after decontamination using autoclave.

**Fig 5:**
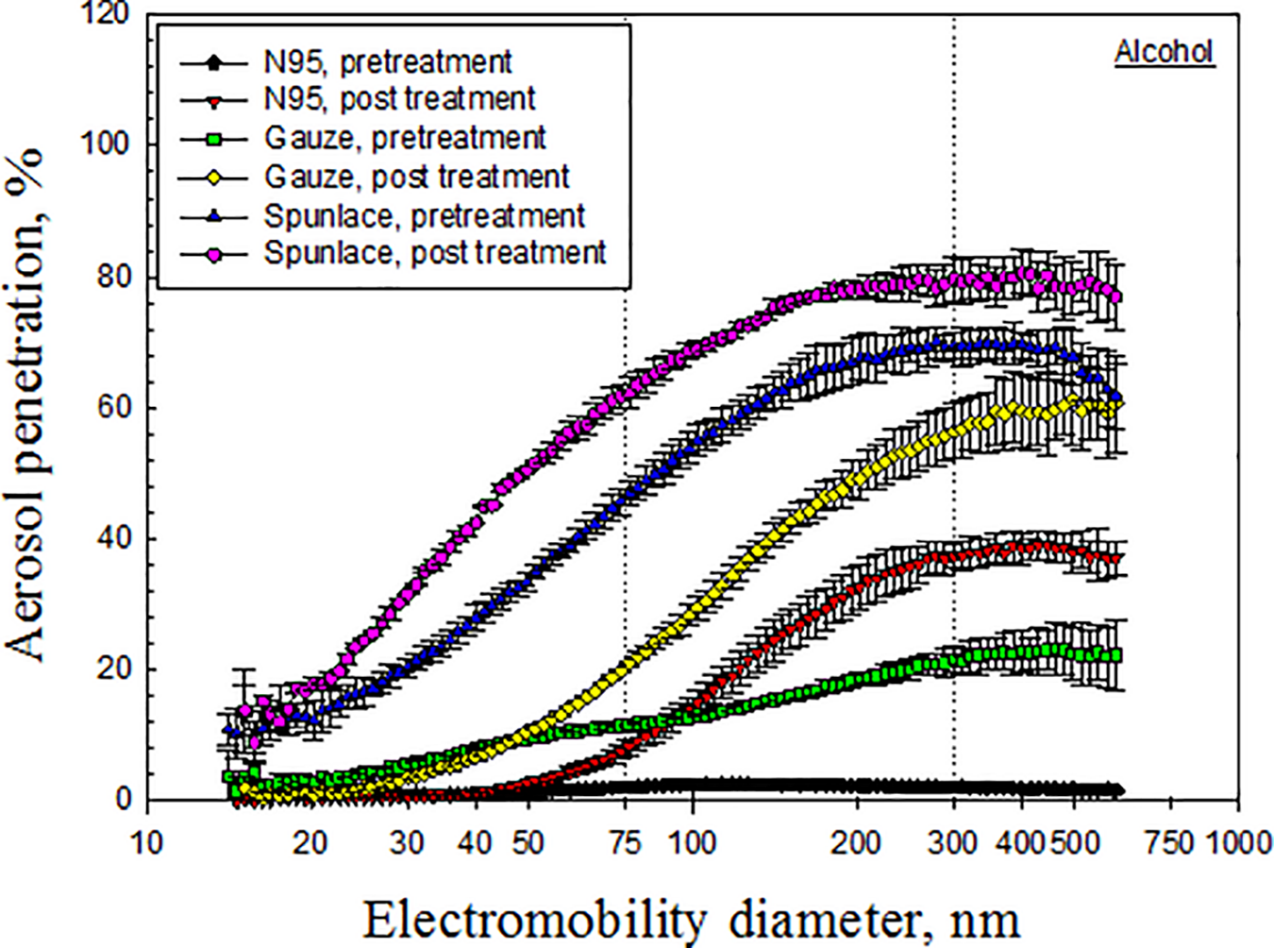
Penetration of particles through mask after decontamination using ethanol.

**Fig 6:**
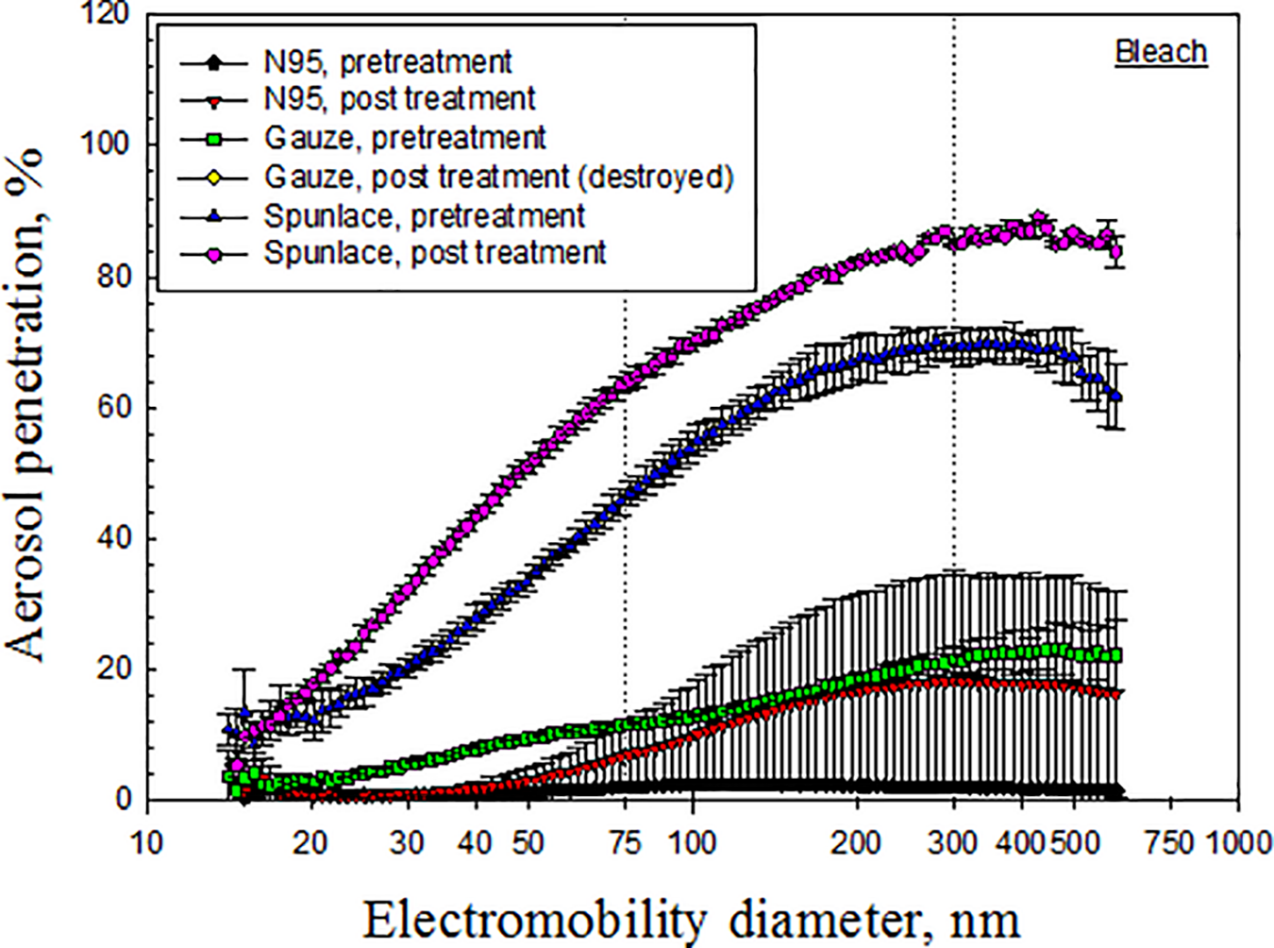
Penetration of particles through mask after decontamination using bleach.

**Fig 7:**
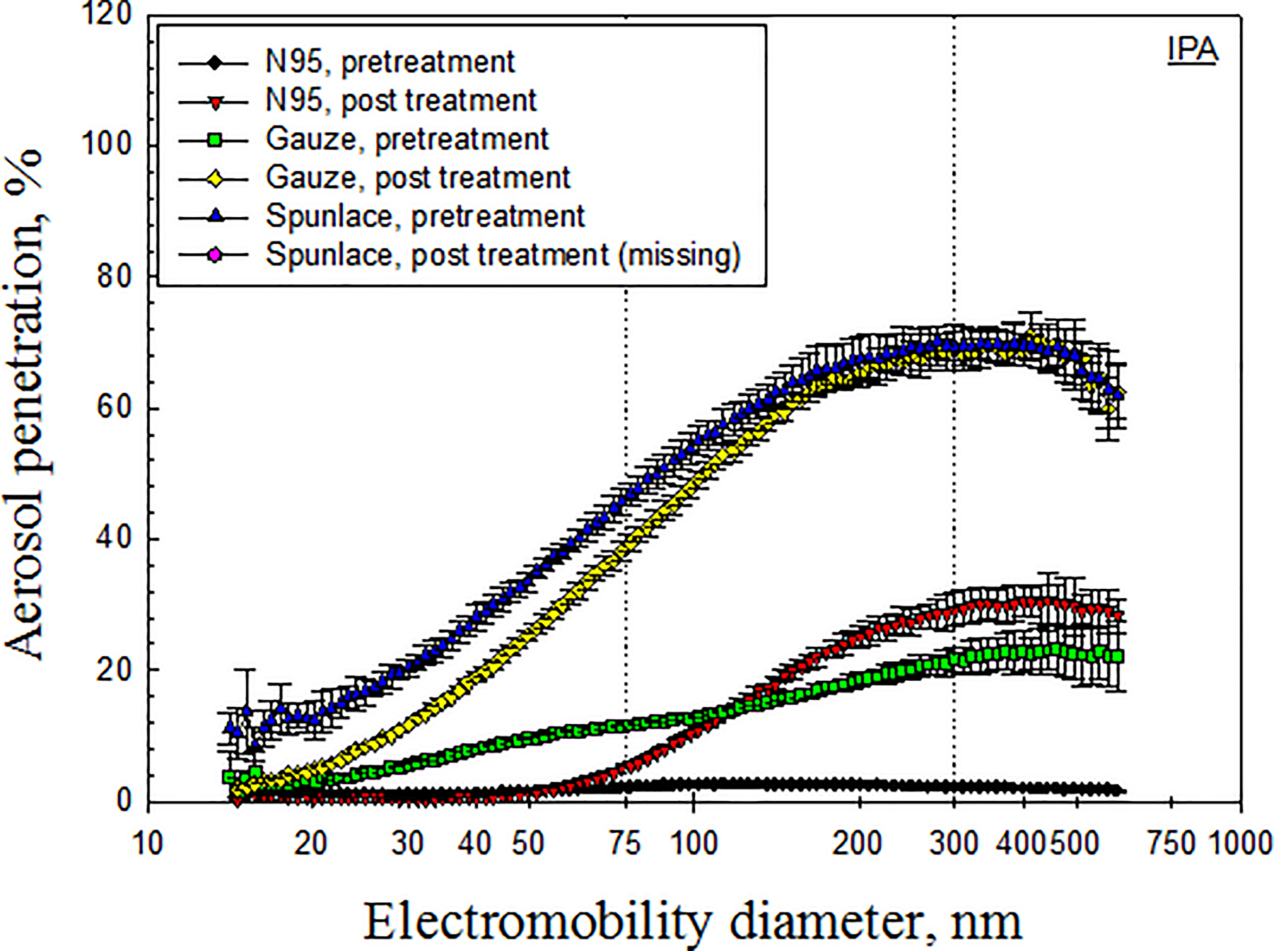
Penetration of particles through mask after decontamination using IPA.

Figs 3–7 depict the penetration of particles through the electret masks before and after decontamination using the five selected methods. Before decontamination, the particle penetration through the N95 mask did not exceed 5%. However, the penetration of particles larger than 27.9 nm through the Gauze mask exceeded 5%, and that of particles from 14.1 nm to 594 nm through the Spunlace mask exceeded 8.6% (Fig 3). According to Fig 3, the penetration of particles through the test masks following heating in the rice cooker was similar to that before heating. Treating the masks in an autoclave increased the penetration of particles through the Spunlace and Gauze masks (Fig 4). Figs 5–7 reveal that treating the test masks with ethanol, bleach or isopropanol raised the penetration of particles, (especially larger ones. Submersion in bleach significantly changed the penetration through the N95 masks (Fig 6).

Electret fibrous filters, which were used in the masks were tested in this investigation, are highly representative of such respirators. For a given fiber diameter, packing density, and thickness, electret filters have much higher filtration efficiency than non-electret filters, particularly for submicron aerosols [12]. To assess the effect of charge density on filtration efficiency, filters were dipped in isopropanol to remove charges before measuring the filter quality [2]. The charge density of filters strongly affected the filtration efficiency. Reducing the charge density increased aerosol penetration. Treatment of N95 masks with isopropanol increased the penetration of particles larger than 50 nm, owing to a reduction in the charge density of the filter.

The experimental results indicate that decontamination by dry heat in a rice cooker had little effect on the penetration of particles through any of the tested masks (Fig 3), while wet heat in the autoclave had little effect on that of the N95 mask (Fig 4). From a previous study [8], the penetration of N95 masks that were sealed in a standard poly/paper autoclave bag and then treated in an autoclave, as measured using a TSI Model 8130 automated filter tester, increased from 0.7% to 18.7% (*p* = 0.003). However, in the same study, the penetration of particles had little effect on the N95 masks that were treated with tap water (*p* = 0.417) or dry heat at a temperature of 80°C (*p* = 0.192). The major discrepancies of penetration of particles between pretreated and treated masks were discussed in the later section of MPS.

Chemical methods that involved submersion in liquid ethanol, isopropanol or bleach influenced the penetration of particles through the electret masks, probably because alcohols and bleach eliminated the electrostatic charges on the filters [22], and characteristics such as fiber diameter, packing density and charge density determined the ultimate effect [12]. Bleach destroys the structure of Gauze filters. Treatment with bleach seriously damaged the structure of the filter in the double-layer static Gauze mask used in this study, preventing it from being tested further (Fig 6). Similarly, FFR melted under microwave irradiation or dry heat starting at 100°C. [9].

**Fig 8:**
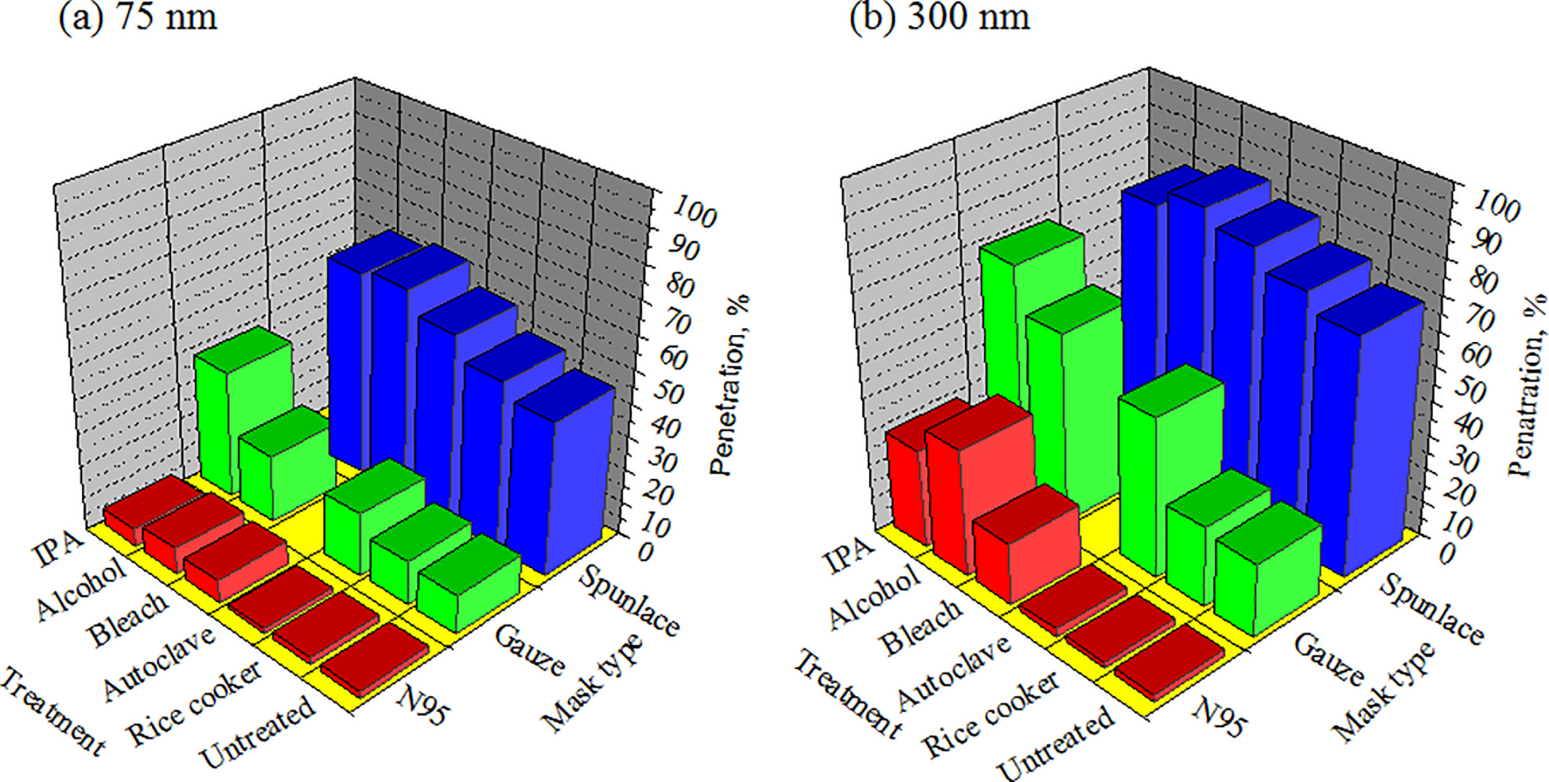
Penetration of 75nm and 300nm particles through mask. * indicates *p*<0.05.

The penetrations of particles before and after decontamination were experimentally compared using particles of traditional standard sizes for penetration tests. The standard sizes were 75 nm [10] and 300 nm for electret masks and mechanical filter filtration [12], respectively. The results thus indicate that decontamination increased the penetration of both 75 nm and 300 nm particles through the masks (*p*<0.05), except in the cases of decontamination of N95 masks in a rice cooker or an autoclave, and Gauze and Spunlace masks in a rice cooker (Fig 8). Moreover, Spunlace masks treated by ethanol did not statistically significant increase the penetration of 300 nm particles (*p* = 0.093).

### Most penetrating particle size (MPS)

The experimental MPS values of the pretreated N95, Gauze, and Spunlace masks were 118 nm, 461 nm and 279 nm, respectively (Table 2). All of the MPS values of the pretreated masks exceeded 75 nm, and that of the Gauze masks exceeded 300nm. The MPS value of the N95 masks increased upon decontamination, except when using the rice cooker (Table 2). Most of the MPS values following decontamination exceeded 300 nm, except for N95 treated with dry heat using the rice cooker, for which the MPS remained at 118 nm, and the penetration at 118 nm remained approximately 2.6%. The MPS value was 346 nm for autoclave-treated N95 masks and larger than pretreated ones (118 nm). These results are comparable and only matches to an earlier finding condition that aerosol penetration decrease and MPS increase with decreasing face velocity (mechanical filter) [22]. However, autoclave is a common sterilization method and further study on FFRs is necessary.

**Table 2:** Most penetrating particle sizes (MPS, nm), associated penetration (*P*, %) and direction of change in pressure drop (Δ*p*).

Treatment	N95		Gauze		Spunlace	
	MPS(P)	Δp*	MPS(P)	Δ*p*	MPS(P)	Δ*p*
Pretreatment	118 (2.6)		461 (23.2)		279 (70.0)	
Rice cooker	118 (2.5)	--	445 (24.9)	--	445 (77.1)	--
Autoclave	346 (2.4)	**↓**	478 (49.8)	**↓**	429 (81.6)	--
Ethanol	445 (39.0)	--	496 (61.4)	--	400 (80.7)	--
IPA	445 (30.7)	NA	414 (71.1)	NA	NA	NA
Bleach	300 (18.3)	**↑**	NA	NA	429 (89.0)	--

*:--similar; ↑ increase; **↓** decrease.

NA: data not available.

The MPS value following immersion in bleach was 300 nm, satisfying the requirements for a mechanical filter [12]. The MPS of the decontaminated Gauze was between 414 nm and 496 nm. Decontamination increased the MPS of Spunlace to more than 400 nm. These results are consistent with an earlier finding that both aerosol penetration and MPS increase with decreasing charge density [22].

### Effect of decontamination on pressure drop through masks

**Fig 9:**
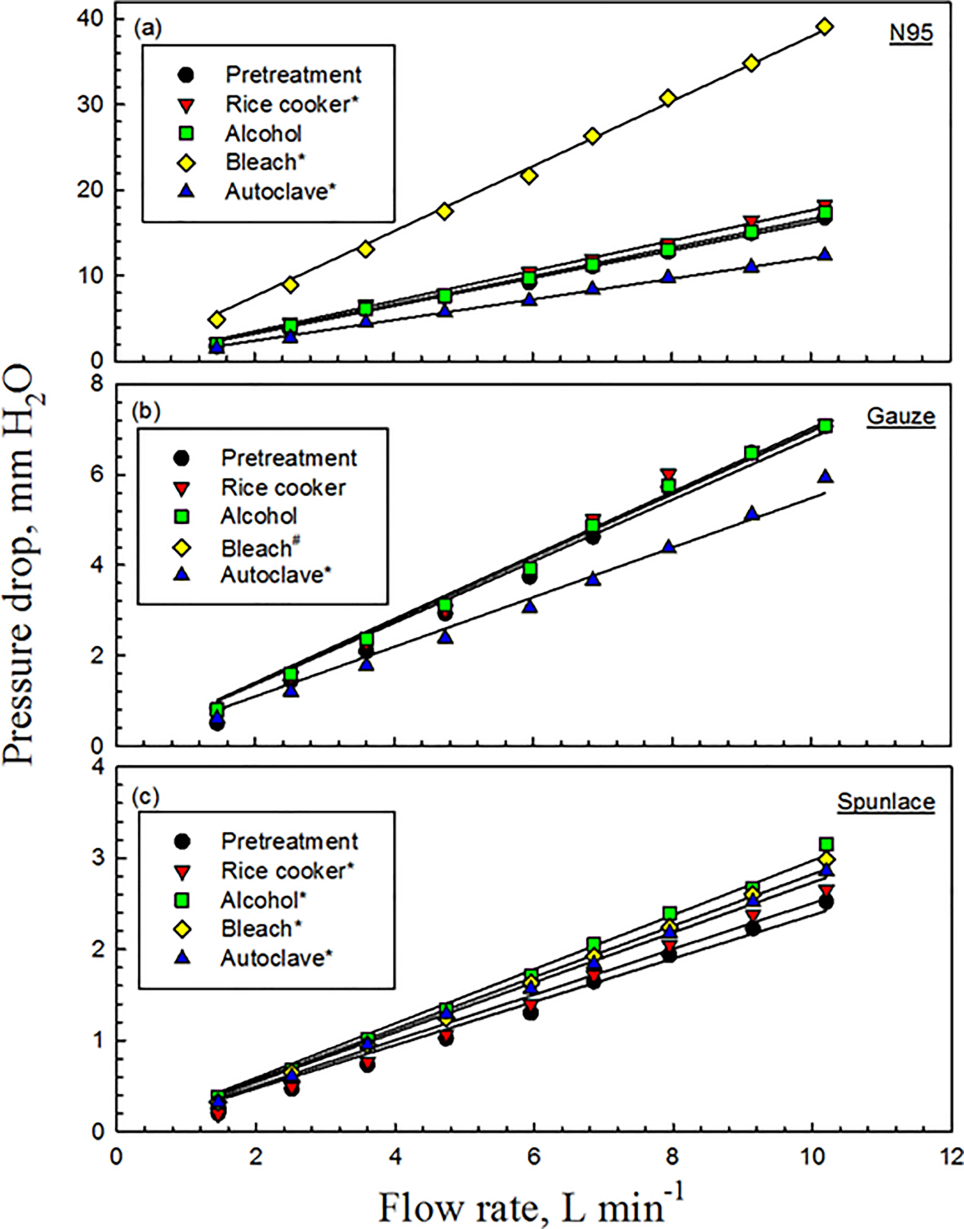
Pressure drop through mask before and after decontamination. * indicates *p*<0.05; # indicates the Gauze masks were destroyed and therefore their Δ*p* could not be obtained.

For the N95 masks before decontamination, Δ*p* increased from 1.7 mm H_2_O to 16.8 mm H_2_O as the flow rate increased from 1.5 L min^−1^ to 10.2 L min^−1^; decontamination using ethanol produced similar results (*p* = 0.869) (Fig 9(A)). For the N95 masks submerged in bleach for 10 min, Δ*p* linearly increased from 4.9 mm H_2_O at 1.5 L min^−1^ to 39.2 mm H_2_O at 10.2 L min^−1^ (*p*<0.001). Comparable results were found when using rice cooker (*p* = 0.019). However, the Δ*p* of the autoclaved N95 masks fell (*p*<0.001). The measured values of Δ*p* (9.2 mm H_2_O) at a flow rate of 5.95 L min^−1^ were less than 35 mm H_2_O, which is the upper limit for inhalation as specified in 42 CFR 84 subpart K [10].

For the Gauze masks before decontamination, Δ*p* increased from 0.5 mm H_2_O to 7.1 mm H_2_O as the flow rate increased from 1.5 L min^−1^ to 10.2 L min^−1^. Similar results were obtained following decontamination using the rice cooker (*p* = 0.893) or ethanol (*p* = 0.201) (Fig 9(B)). When the Gauze masks were decontaminated using the autoclave, Δ*p* linearly fell from 0.6 mm H_2_O at 1.5 L min^−1^ to 5.9 mm H_2_O at 10.2 L min^−1^ (*p*<0.001). However, the Gauze masks were destroyed when they were submerged in bleach, and therefore their Δ*p* could not be obtained.

For the Spunlace masks before decontamination, Δ*p* increased from 0.2 mm H_2_O to 2.5 mm H_2_O as the flow rate rose from 1.5 L min^−1^ to 10.2 L min^−1^, and sharper slopes were obtained following treatment by a rice cooker, ethanol, bleach, and an autoclave (*p*≤0.01) (Fig 9(C)).

The flowrate through the filter influences pressure drop. Thus, masks of all three types exhibited a linear relationship between pressure drop and flowrate following the application of each of the decontamination methods. Immersion in bleach increased the pressure drop through the N95 mask (to 40 mm H_2_O at a flowrate of 10 L min^−1^), whereas use of the autoclave reduced it. Similar results were obtained in the tests on the Gauze masks except when bleach was used, because bleach destroyed all of the masks.

**Fig 10:**
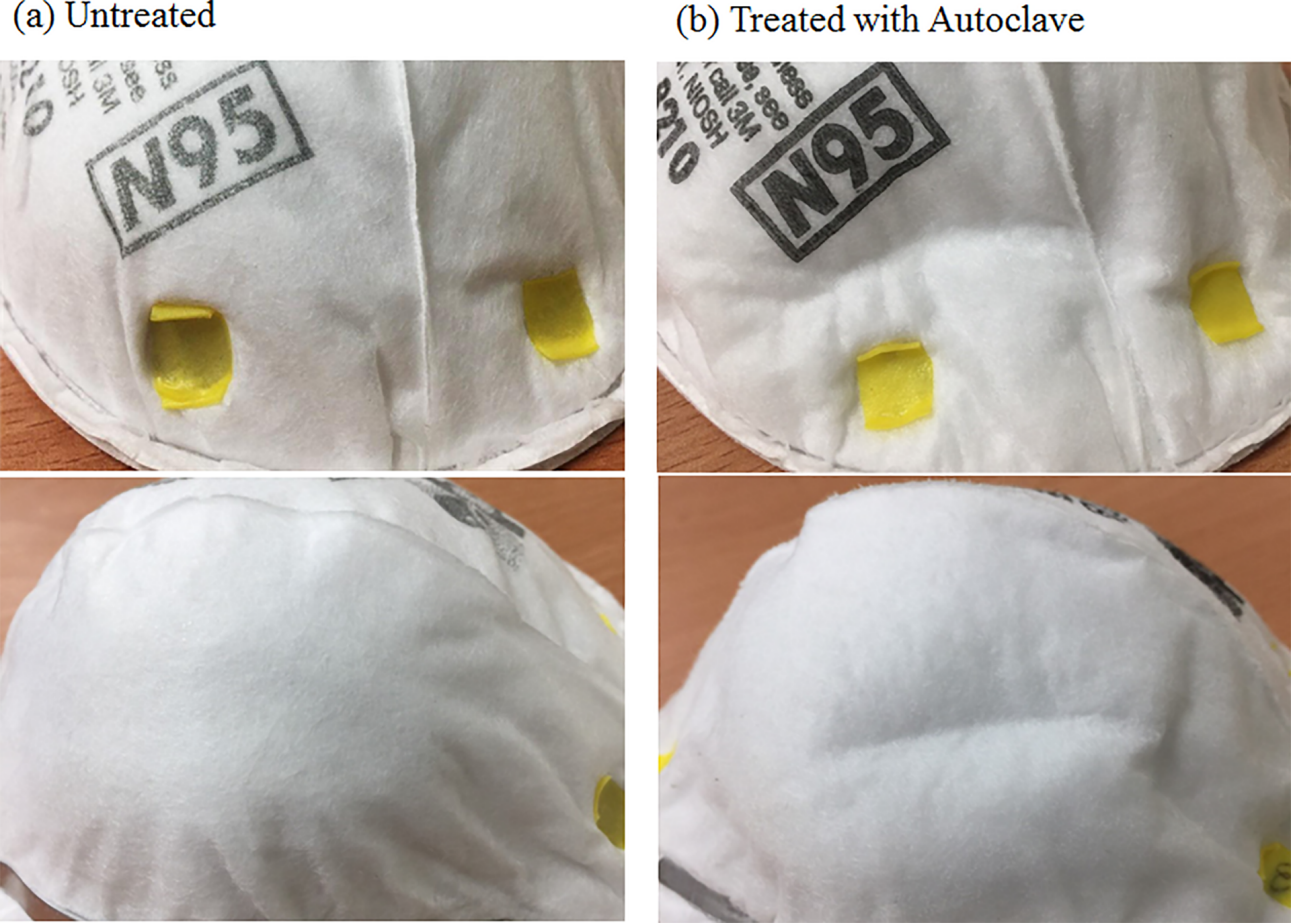
Visual changes of N95 masks after using an autoclave.

Decontamination typically increased Δ*p*, but treatment in the autoclave did not do so for the N95 or Gauze masks. The low pressure drop results for these two types of masks were observed to be associated with folds of the inner and outer filter supports. These folds may reduce the uniformity of filter density, causing the air to flow through loose filter material in the mask, and reducing the resistance of the filter. From the observation, as show in Fig 10, the outer layer of the masks were deformed, shrunken and stiff and these phenomena were similar to a previous study [8]. But no remarkable mottle was observed. However, the characteristics of various filters are not easily obtained as this information is commercially sensitive, and this fact imposed a limitation on this study.

Moreover, in the study, the calculated inspiratory breathing resistance (IBR) at 85 L min^-1^ [23] was 6.5, 2.6 and 0.9 mm H_2_O/L/sec for the pretreated N95, Gauze and Spunlace masks, respectively. Previously reported maximum value for the normal threshold for detection of IBR was 7.6 mm H_2_O/L/sec [23]. In the study, the highest IBR value was 15.3 mm H_2_O/L/s for the N95 masks submerged in bleach which was the only one exceeded 7.6 mm H_2_O/L/sec. All the other IBR values of treated masks ranged from 1.0–7.4 mm H_2_O/L/s. The pressure drop probably playing a role if inhalation resistance exceeded 35 mm H_2_O (equal to 24.7 mm H_2_O/L/s), but since few of the masks came close to that, the primary concerning factor of *q*_*f*_ should focus on filter penetration.

### Filter quality before and after decontamination

**Fig 11:**
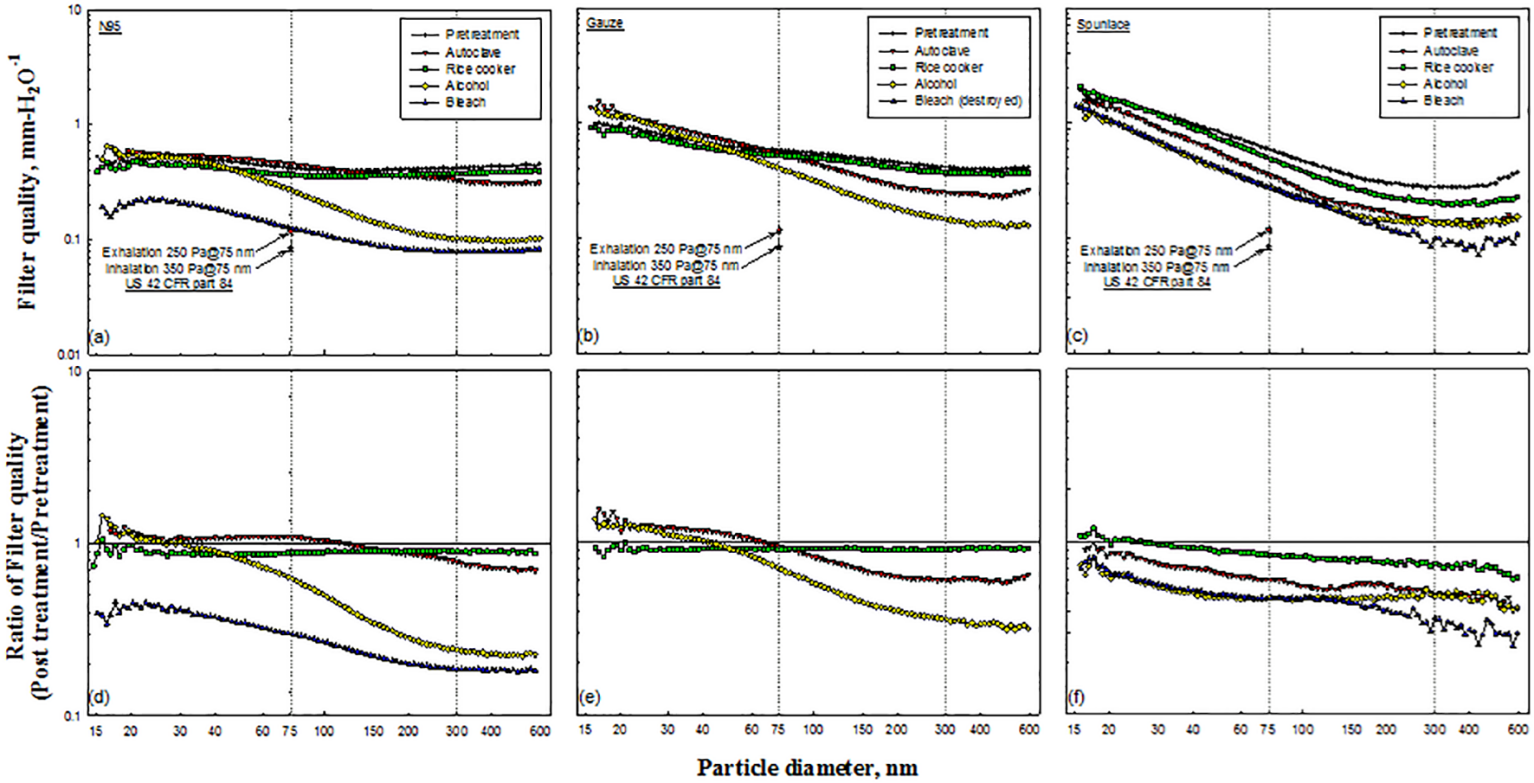
Filter quality of tested masks and corresponding ratios.

The *q*_*f*_ values in this work were computed from the penetration of particles and the pressure drop through the filter. Most calculated *q*_*f*_ values were between 0.1 and 1, and this parameter was found to be a function of particle diameter (Fig 11(A)–11(C)). The highest values of *q*_*f*_ were obtained for smaller particle diameters, and the lowest values were obtained for particle diameters of around 400–500 nm, which was the range of experimental MPS in this investigation (Table 2).

Regulation US 42 CFR part 84 requires that the resistances of particulate respirators upon initial exhalation and inhalation do not exceed 25 mm H_2_O and 35 mm H_2_O, respectively [10], and the calculated *q*_*f*_ values are 0.117 mm H_2_O^−1^ and 0.084 mm H_2_O^−1^, respectively (Fig 11(A)–11(C)). All of the values of *q*_*f*_ that were obtained in the test exceeded the calculated values, indicating that all of the masks satisfied the calculated *q*_*f*_ values (0.117 mm H_2_O^−1^ and 0.084 mm H_2_O^−1^) after decontamination. However, *q*_*f*_ incorporates two variables, namely aerosol penetration and pressure drop. The minimum filtering efficiency (≥95% for N95, for example) should be considered before obtaining *q*_*f*_.

Figs 11(D)–11(F) present the calculated ratios of *q*_*f*_ values before and after decontamination. Most of the *q*_*f*_ ratios of the N95 masks were less than 1.0, except when treatment involved the autoclave or ethanol (Fig 11(D)). The pressure drop (Fig 9(A)), which is the denominator in *q*_*f*_, decreased when decontaminating N95 masks using the autoclave. Decontaminating N95 masks using ethanol did not affect the penetration of particles with diameters of less than 33.4 nm (Fig 6), and yielded a *q*_*f*_ ratio of close to unity. Under the Boltzmann charge equilibrium, approximately 60% of particles with diameters of less than 50 nm were uncharged [15].

Similar results were observed in the test involving the Gauze mask (Fig 11(E)). Most of the *q*_*f*_ ratios for Spunlace masks were less than 1.0 (Fig 11(F)). In most of the tests, the ratio of *q*_*f*_ values decreased as the particle size increased.

### Overall filter quality, *q_f,o_*

Since *q*_*f*_ is a function of particle size, *q*_*f*,*o*_ was calculated using Eq (3), in which the value of *q*_*f*_ is weighted by particle size ranging from 14.6 nm to 594 nm. Table 3 presents the relevant results, including the full range of sizes and *q*_*f*,*o*_ values for particles that are smaller and larger than 100 nm (PM_0.1_, PM_0.1–0.6_), and the ratio of *q*_*f*,*o*_ values before and after decontamination. The values of *q*_*f*, *o*_ for pretreated N95, Gauze and Spunlace masks over the full range of particle sizes were 0.69 mm H_2_O^−1^, 0.92 mm H_2_O^−1^, and 1.07 mm H_2_O^−1^, respectively. The pretreated Spunlace masks had the highest *q*_*f*,*o*_ because they had the lowest pressure drop (Fig 9). Before decontamination, the N95 mask had the lowest *q*_*f*,*o*_ over the full range of particle sizes and for PM_0.1–0.6_. The factor that most strongly influenced these results was the pressure drop, which was highest for the N95 mask (Fig 9). The Spunlace mask had the lowest value of *q*_*f*,*o*_ for PM_0.1–0.6_, because it had the highest penetration (Figs 3–6).

**Table 3:** Overall filter quality and ratio of values before and after decontamination.

Size range	Pretreatment	Autoclave	Rice cooker	Ethanol	Bleach
N95					
Full*	0.69(1.00)	0.67(0.98)	0.61(0.89)	0.42(0.61)	0.20(0.33)
PM_0.1_	0.36(1.00)	0.40(1.10)	0.32(0.88)	0.33(0.90)	0.14(0.37)
PM_0.1–0.6_	0.32(1.00)	0.27(0.84)	0.29(0.90)	0.10(0.30)	0.07(0.20)
Gauze					
Full*	0.92(1.00)	0.90(0.98)	0.84(0.91)	0.73(0.79)	NA
PM_0.1_	0.57(1.00)	0.67(1.17)	0.52(0.91)	0.58(1.02)	NA
PM_0.1–0.6_	0.35(1.00)	0.23(0.66)	0.32(0.91)	0.14(0.41)	NA
Spunlace					
Full*	1.07(1.00)	0.75(0.70)	0.98(0.91)	0.58(0.54)	0.58(0.54)
PM_0.1_	0.82(1.00)	0.62(0.76)	0.79(0.97)	0.46(0.57)	0.48(0.59)
PM_0.1–0.6_	0.25(1.00)	0.13(0.52)	0.19(0.75)	0.12(0.47)	0.10(0.38)

*: from 14.6 to 594 nm

NA: data not available.

Decontamination decreased all values of *q*_*f*,*o*_ over the full range of particle sizes (Table 3). The ratios of *q*_*f*,*o*_ values ranged from 0.33 (for N95 immersed in bleach) to 0.98 (for autoclaved N95 and Gauze masks). Similar results were obtained for PM_0.1–0.6_, for which the ratios ranged from 0.2 (N95 immersed in bleach) to 0.91 (Gauze mask in the rice cooker). However, for PM_0.1_, *q*_*f*,*o*_ increased upon treatment in three cases, which were the autoclaved N95, the autoclaved Gauze masks and the ethanol-immersed Gauze masks. The N95 immersed in bleach had the lowest *q*_*f*,*o*_ value. In all tests, *q*_*f*,*o*_ for PM_0.1_ exceeded that for PM_0.1–0.6_ because particles smaller than 100 nm penetrate less, reflecting higher filtration quality for a given pressure drop. The *q*_*f*,*o*_ values indicate that physical decontamination methods are better than chemical methods, especially for PM_0.1_.

A good respirator should have a high overall value of *q*_*f*_, including low aerosol penetration and a low pressure drop. Basically, Table 3 would suggest that Gauze and Spunlace masks are the best because they have a higher *q*_*f*_ than N95 masks due to their low pressure drop. However, respirator selection should prioritize penetration and fit factor and not *q*_*f*_. Neither mask in the study meets criteria for penetration despite their high *q*_*f*_. In current study, only the tested N95 masks matched the minimum 42 CFR Part 84 criteria [10] because they had both an acceptable pressure drop and penetration. In 2011, Viscusi et al. [24] demonstrated that incubation in moist heat significantly reduced the fit factor of two of the six FFRs (*p* < 0.05). For these two FFRs, fit factors after decontamination remained ≥100. In our study, neither *q*_*f*_ nor *q*_*f*,*o*_ consider fit factor when considering mask performance. Since the fit factor importantly affects the effectiveness of a worn mask, the end users should also consider the effect of decontamination on fit. This investigation did not evaluate the bioefficacy of each FFR following treatments. Our research group will publish results on these topics elsewhere.

## Conclusion

Decontamination increases the MPS, and changes the *q*_*f*,*o*_ of N95, Spunlace and Gauze masks. Filter quality decreased as the particle size increased due to an increase in penetration. Decontamination increased the pressure drop, except for N95 and Gauze masks that were decontaminated using an autoclave. Decontamination reduced the value of *q*_*f*,*o*_, unless performed by using an autoclave or rice cooker, which created observable folds of the masks.

Instruments currently used for measuring particle size distributions can be used to measure the *q*_*f*,*o*_ over the full range of particle sizes in this study, in order to select decontaminated masks. However, different particle sizers can be used with different size ranges, so calculated values of *q*_*f*,*o*_ ought to be associated with corresponding PSDs. Moreover, a particle sizer with a size range that better covers the MPS of the test masks should be used to study the effect of particle size on *q*_*f*,*o*_.

The Spunlace mask had a higher *q*_*f*,*o*_ value than the N95 FFR, and seemed to perform better, but it provided minimal protection because of the high penetration. Consequently, buyers of FFRs or masks must consider both the penetration and the pressure drop. A good respirator should have a low aerosol penetration as the most important characteristic. Since the breathing resistance of N95 is 9.2 mm H_2_O at 5.95 L min^−1^, the wearer may not notice a difference in pressure drop after decontamination. Therefore, for N95 respirators with a pressure drop below a 35 mm H_2_O threshold, the aerosol penetration must be kept below 5% consistent with 42 CFR 84 subpart K [10]. Moreover, it should be noted that the calculation of *q*_*f*_ and *q*_*f*,*o*_ provides more weight on pressure drop for comparing respirator after decontamination, therefore, *q*_*f*_ and *q*_*f*,*o*_ should be used carefully.

## Supporting information

S1 File Penetration & pressure drop. This is the data of Fig 3 to Fig 7.
(XLSX)
